# A Narrative Review of Alternative Protein Sources: Highlights on Meat, Fish, Egg and Dairy Analogues

**DOI:** 10.3390/foods11142053

**Published:** 2022-07-11

**Authors:** Miguel Lima, Rui Costa, Ivo Rodrigues, Jorge Lameiras, Goreti Botelho

**Affiliations:** 1 Polytechnic Institute of Coimbra, Coimbra Agriculture School, Bencanta, 3045-601 Coimbra, Portugal; limamiguel15@gmail.com (M.L.); ruicosta@esac.pt (R.C.); ivorod@esac.pt (I.R.); jorge.lameiras@esac.pt (J.L.); 2 Research Centre for Natural Resources, Environment and Society (CERNAS), Coimbra Agriculture School, Bencanta, 3045-601 Coimbra, Portugal

**Keywords:** meat analogue, plant-based protein foods, food engineering, sustainability

## Abstract

The research and development of alternatives to meat (including fish) and dairy products for human consumption have been increasing in recent years. In the context of these alternatives, there is a diversity of products such as tofu, tempeh, seitan, pulses, algae, seeds, nuts and insects. Apart from these, some products require new technical processes such as needed by milk drink alternatives, mycoprotein and meat, cheese and fish analogues. The aim of these analogues is to mimic the physical and organoleptic properties of animal origin products through fibrous composition and mix of ingredients from vegetable sources using adequate technology, which allow providing similar texture and flavor. Using a narrative approach to review literature, the objectives of this paper are to systematize the arguments supporting the adoption of meat, eggs and dairy alternatives, to identify the diversity of alternatives to these products on the market, including the related technological processes, and to project the challenges that the food industry may face soon. From a total of 302 scientific papers identified in databases, 186 papers were considered. More research papers on products associated with alternatives to milk were found. Nevertheless, there are products that need more research as analogues to meat and dairy products. A general scheme that brings together the main reasons, resources and challenges that the food industry faces in this promising area of alternatives to meat and dairy products is presented.

## 1. Introduction

Meat consumption has been, for a long time, considered an essential component of human nutrition. However, the considerable increase in production associated not only with the increase in the human population but also with generally excessive consumption of meat on a global scale [1] has given rise to concerns of environmental, public health and ethical and ideological nature. The reasons pointed out by researchers as alarming in terms of the environment are inadequate management of water resources and arable land, the emission of harmful gases into the atmosphere [2], the reduction in biodiversity [3] and the harmful effects of the use of antibiotics and other medicines in livestock and agriculture [4]. In terms of public health, the epidemiological relationship between the consumption of red meat and processed meat with some pathologies, such as colon cancer [5] and cardiovascular problems [6], is a matter of concern. In fact, there is a broad set of plant origin food traditionally used in the human diet worldwide, such as in the Mediterranean Diet, representing important food sources of protein and associated with the maintenance of good health levels. Those mentioned deleterious consequences on human health represent an incentive to reduce the consumption of products of animal origin, reinforced by the growing ethical concern with the welfare of animals [7] and general environmental sustainability. The food industry has shown the capability to rapidly adapt and innovate to meet the growing demand for more sustainable diets. This initiative is particularly reflected in the growing market for alternative proteins, which are increasingly becoming available to consumers. Alternative protein sources encompass everything from algae to re-engineered plant-based products, innovative use of legumes and a variety of meat substitutes. Nowadays, there is a large range of possibilities available in the market: lab-grown meat, plant-based meat, single-cell proteins from yeast or algae, and edible insects.

To reduce the consumption of proteins of animal origin, several strategies were created, such as: the encouragement to define a day when meat consumption is substantially reduced [8]; the appeal for the replacement of the consumption of meat for proteins of vegetal origin such as beans, nuts and/or legumes; and the development of meat-like products [9]. This paradigm sustains a tendency to search for alternatives to products of animal origin, which requires the use of new technology, especially in meat analogues and vegetable drinks. The use of these technologies makes it possible to bring the functional, organoleptic and nutritional properties of meat analogues closer to products of animal origin, through the processing of fibrous material from ingredients of plant origin, in order to imitate the muscle tissue texture [10]. Several techniques are used, the most used being extrusion, electrospinning and wet spinning. However, in order to reduce the consumption of proteins of animal origin by replacing them with alternatives to meat, it will be necessary to overcome barriers such as resistance to change due to the high status of animal origin products represent for the consumer, as well as the established economic interests in the value chain and the need to deepen technological knowledge for meat analogue processing [11]. The increasing demand for protein has resulted in rapid innovations devised by the food industry in categories such as alternative proteins, for which nutritional content can still be improved.

Using a narrative approach to reviewing literature, the objectives of this paper are to systematize the arguments supporting the adoption of meat and dairy alternatives, to identify the diversity of alternatives to these products on the market, including the related technological processes, and to project the challenges that food industry may face in near future. To fully understand the characteristics of the alternatives to meat and dairy products on the market, an overview of the main technological processes used in the production of meat and dairy analogues and their basic chemical properties is provided. In this context, meat, fish and dairy analogues, as well artificial meat, mycoprotein and vegetable drinks, are included.

## 2. Materials and Methods

From a methodological point of view, a search was carried out in the b-on, PubMed^®^, Science Direct databases and websites of international organizations such as the Food and Agriculture Organization (FAO) and the World Health Organization (WHO), without limitation of date or origin of studies. The keywords used were “meat analogues”, “vegetable protein” and “meat alternatives”, and 302 articles were identified with the potentially relevant title. Of these, and after partial (only the abstract) or full reading, 186 articles were considered in the present narrative review. Narrative review has been frequently used by several authors [12]. Additionally, books by an international publisher and research on websites of some national and international organizations were considered. This article begins by specifically identifying alternative products to those of animal origin, from the oldest ones (tofu, tempeh, seitan, algae, legumes, insects) to the products that are more similar to meat (artificial meat, mycoprotein, fish alternatives) as well as vegetable drinks. Additionally, an analysis of the technologies commonly used to produce these products is presented, as well as an individual analysis that identifies the products, which describes the production processes and contextualizes with the nutritional information of each category of products.

## 3. Meat and Dairy Alternatives

A diverse array of alternative foods to meat and dairy products is currently available on the market. There are long-established products such as tofu, tempeh and seitan, seeds, legumes, beans and nuts, and others such as algae and insects. Complementarily, there are products trying to resemble meat, which are products that aim to replace products of animal origin, imitating their functional properties and replicating their organoleptic characteristics. Table 1 lists the main categories of alternatives to animal origin products, mainly based on review papers published worldwide between 2014 and 2021.

In general, the reviewed papers published from 2014 until 2021 detail the composition of the products and identify the functional properties of each ingredient in the production of a meat analogue. A meat analogue product, in its general composition, contains water (50–80%), textured vegetable protein (15–20%) and non-textured protein (10–25%), flavorings (3–10%), fat (0–15%), binding agents (1–5%) and colorants (0–0.5%) [28]. Other specific ingredients may be used to improve the texture of the final product.

Table 2 identifies and characterizes the existing products, as well as their advantages and disadvantages, supported by the available literature. In the field of advantages, arguments around the contribution to environmental sustainability are particularly valued. On the other hand, in the field of disadvantages, sensory and legislative aspects stand out. 

It is important to adapt the technological processes of extraction of fibrous tissue from plant-based proteins to the characteristics of foods, to obtain final products of high protein purity. The production of meat analogues requires the use of technologies that allow the mimicking of properties of original animal products. In Table 3, some of these technologies are identified.

### 3.1. Plant-Based Proteins

There is a set of products of plant origin that constitute an important food source of proteins. While individually these products may have limitations in terms of several essential amino acids and vitamin B12, their combination can meet the nutritional needs of a healthy individual [13,29,103]. Table 4 describes the main plant origin protein sources.

### 3.2. Plant-Based Drinks

The production processes of plant-based drinks show some variants depending on the raw materials used. However, the production methods of vegetable drinks share common operations, which are described in Table 5.

Initially, it is required to decide whether or not to peel the selected raw material. This can be purchased already peeled or unpeeled, dry or fresh. If the raw material is purchased fresh and with the peel, it must be placed in hot water to later remove the peel [121]. After peeling, the preparation will need to be dried [122]. On the other hand, if the product is received already dried, it goes directly to a dry roasting or grinding stage.

### 3.3. Mycoprotein

Mycoprotein is a product created from filamentous fungus (*Fusarium venenatum*), used as an alternative to meat [165,166].

The filamentous fungus is produced in reactors through continuous fermentation processes, in which conditions are carefully controlled (e.g., pH and temperature), with subsequent stages being important for molding the product [10]. After fermentation, the RNA must be degraded into monomers through a heat treatment, so that it can diffuse to the outside of the cells. Residual biomass is heated and centrifuged to obtain a slurry with 20% solids [167]. The filamentous fungus is disintegrated after this centrifugation step and, later, other steps follow, such as molding, steaming, cooling and texturing. These steps are necessary to obtain a fibrous product. Mycoprotein is usually mixed with a small amount of egg albumen, a little bit of roasted barley malt extract and water, or a natural flavoring, and blended instead of malt to give it a flavorful character. Nutritionally, its composition is comparable to meat, with proteins of good bioavailability, low fat and high content in fiber [15,23,168].

Commonly, mycoprotein-derived products are marketed as sausages, hamburgers or in small pieces [169]. Although this procedure has been around for decades, it is relatively intensive in the use of energy and ingredients resources [42].

### 3.4. Edible Algae

Algae have been part of the human diet for many years, based on archaeological evidence, predominantly in Asia (traditionally in China, Japan and South Korea). More recently, consumption of seaweed as food has appeared in European coastal areas (e.g., France, Norway, Wales and Ireland) [68]. The difference between algae results from their chemical and morphological structure. Depending on their size, algae can be classified as unicellular microalgae or macroalgae [170,171].

Algae are considered a food rich in proteins and a source of eicosapentaenoic (EPA) and docosahexaenoic (DHA) fatty acids, and their composition varies depending on the species being evaluated [29]. However, digestibility and bioavailability can be disturbing factors, as the cell wall interferes with the use of nutrients. To increase the bioavailability of algae proteins, a pre-treatment may be necessary to help break down the cell wall. Among the advantages of producing algae as food is its ability to fix carbon dioxide and use less soil than the livestock industry, contributing to the preservation of the environment [29]. In addition to these advantages, around 130 scientific and medical institutions have stated that algae products can bring benefits to human health and the environment in the medium term [172]. The most popular algae for human consumption are spirulina and chlorella. It is a low-processing product, subject to dehydration and contains more than 70% protein, including all essential amino acids [69]. Marine species are also used in the production of food additives such as agar−agar, carrageenan and alginates. These substances are used as natural additives as gelling agents, thickeners and stabilizers, respectively [173].

### 3.5. Artificial Meat

In vitro meat is obtained by harvesting cells from live animals, and their subsequent proliferation is performed using cell engineering techniques. This methodology makes the production of meat viable, avoiding large-scale livestock production [174].

The producing process of artificial meat begins with the removal of a small portion of animal tissue, through a biopsy under anesthesia. Subsequently, proliferation takes place, where stem cells are first separated from the original tissues and then developed into other muscle tissue [175,176,177]. Cells are grown in a liquid medium that contains specific nutrients such as amino acids, lipids, vitamins and salts that provide the necessary conditions for tissue development (which may vary depending on cell species and tissue type) [10,178]. The proliferation process doubles the cell population within 7–8 weeks and is a continuous process that takes place in bioreactors until millions of cells are produced. The differentiation phase begins when a sufficient number of cells have been produced and when there are no growth factors in the medium [175]. That said, the cells fuse to form myotubes. The cells are then submerged in a collagen gel with a central hub located in the culture medium to form a muscle fiber in the shape of a donut. The innate ability of muscle cells to contract provides a stimulus for muscle maturation and protein production. As an example, it takes about 10,000 muscle fibers to produce an 85 g hamburger [179].

### 3.6. Alternatives to Cheese

The development of alternative products to cheese involves using fat and/or protein sources that are alternative to that used in conventional products and seeking to simulate the characteristic flavors of cheeses, in a product that will tend to contain lower levels of calories, fat and cholesterol [61]. There are different formulations of alternatives to conventional cheese. For example, there is a formulation that uses caseinates and vegetable oils and a formulation that totally excludes milk and uses plant-based ingredients [180,181]. Among the common ingredients in these products’ processing, acids, flavoring agents and salts are also used.

Table 6 presents the most frequent ingredients in the composition of alternative cheese products.

### 3.7. Alternatives to Fish

Fish consumption is recommended by institutions such as FAO and WHO, due to its high nutritional value [183]. However, excessive consumption has negative consequences on the ecosystem, such as the loss of species biodiversity, environmental damage and diseases of marine species [184,185,186]. In addition, the presence of heavy metals that accumulate in fish due to sea pollution is an added factor that leads to the development of efforts to find alternative solutions [26]. The respective incentives motivate the search for solutions based on plant ingredients that aspire to imitate the characteristics of fish products.

The consumer has a loyalty relationship with conventional products and, therefore, the imitation of sensory properties is considered essential. To achieve that, it is necessary to be able to imitate the intrinsic characteristics of fish products, which requires the simulation of the nanometric fibrous gel structure, resulting from cellular tissues and the organization of protein chains [26]. The common practice to do it is the use of isolated proteins or protein concentrates from vegetable sources such as peas and soy, transformed into “surimi” gels, through partial or total replacement of the raw material of fish or myofibrillar proteins of fish [187,188,189].

In addition to the aforementioned products, there are records of the use of legumes (chickpeas), pseudo cereals (quinoa, buckwheat), wheat (gluten), rice, tubers (potatoes), seeds and nuts in the formulation of fish analogues [24,40].

### 3.8. Egg Analogues

For egg analogues, it is critical to identify a combination of plant proteins that unfolds and aggregates over a similar temperature range as egg proteins (i.e., around 63 to 93 °C), that produces a similar texture−temperature profile, and that produces a similar final appearance and texture [66]. Several plant proteins are able to form gels when they are heated above their thermal denaturation temperatures, such as pea, chickpea, bean, soybean and sunflower, but the protein structure, protein concentration, pH, salt and temperature conditions must be carefully controlled [66]. Egg analogues may also require several other ingredients to simulate specific attributes of real eggs. An emulsified plant-based oil may be included to provide desirable optical, textural, mouthfeel and flavor properties. These oil droplets simulate some of the functional attributes normally provided by the lipoproteins in real eggs. Natural pigments, such as curcumin or carotenoids, are typically added to mimic the desirable yellowish color of the egg. A diversity of flavorings may also be added to obtain an egg-like aroma and taste, such as sugars, salts, herbs, or spices [66].

### 3.9. Edible Insects

Entomophagy is an ancestral custom prevalent in Southeast Asia, the African continent and South America. In western countries, consumption is lower due to the existing cultural bias regarding insects as food [73].

The use of insects as food has the potential to solve problems related to the inefficient use of water resources and topsoil. In this sense, academic institutions, industries and government institutions have made efforts in an attempt to reduce the negative perception of insects through the development of palatable processing methods, as well as to spread the message of the benefits of insect consumption [73].

The main benefits of insects as a food option are the protein content, capable to meet human needs and the high production efficiency compared to other conventional food groups, such as meat. However, insect proteins have low digestibility due to the presence of chitin, which gives them rigidity and makes them resistant to hydrolysis by digestive enzymes. Consequently, insoluble precipitates can be formed, which reduce the bioavailability of minerals and decrease the digestibility of proteins in the small intestine [190]. Furthermore, the presence of high levels of hydrophobic amino acids provides a low solubility and limits the use of insect proteins in food applications.

Table 7 presents the most consumed insects globally according to the existing literature. The edible insects legally allowed to be sold can vary among countries. For example, in European Union, according to the European Food Safety Authority (EFSA), the so-called insect species *Acheta domesticus*, *Alphitobius diaperinus*, *Apis mellifera*, *Gryllodes sigillatus*, *Locusta migratoria* and *Tenebrio molitor* can be produced, marketed and used in human foodstuffs.

The processes used in the industry are strategically outlined in view of the difficulty caused by the negative perception of the consumer regarding this type of products, which encourages opting for processing methods that transform the insects into powder or flour. In this way, it is possible to minimize visual associations and increase palatability and consequent acceptance [191]. In addition, researchers, according to the existing literature, have investigated the functional properties of insect proteins, including gelling ability, foaming ability, emulsifying ability and solubility [73,191].

## 4. Final Remarks and Future Trends

**Figure 1:**
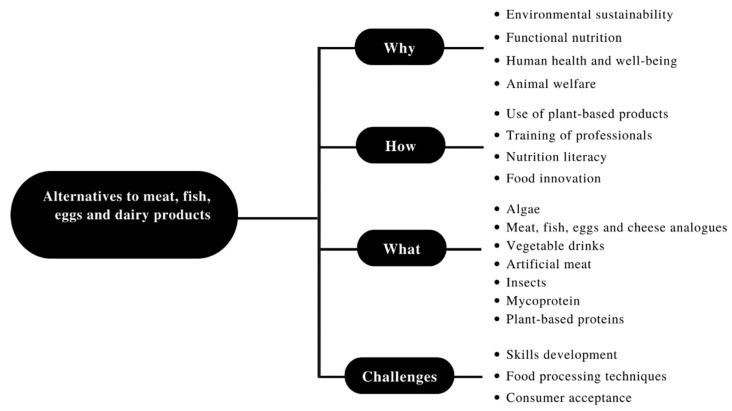
Outlook about main issues related to alternatives to meat, fish, eggs and dairy products in the food industry.

With the purpose of systematizing the main themes focused on in this work, a scheme was built (Figure 1). Fundamentally, this framework, based on the reviewed literature, aims to answer the why, the how and the what and take a forward-looking approach to potential challenges that the food industry may face.

Some topics that were not deeply discussed in this paper, such as training of professionals, nutrition literacy, and consumer cultural acceptance, are included in Figure 1 because they are also closely related to these large thematics around alternatives to meat and dairy products in the food industry. In particular, a broad investment in the nutrition literacy of consumers and in the education of health professionals is essential to promote change in dietary choices.

In terms of public health, the epidemiological relationship between the consumption of red meat and processed meat with some pathologies and the potential of alternatives to meat, such as plant-based diets, for the prevention of chronic diseases and to improve people’s health and quality of life is of utmost importance in public health policies directed to healthy diets, alongside sustainable and inclusive economic growth. It even reinforces the health and nutritional value of some traditional food practices as the high content of vegetables in the Mediterranean diet or the consumption of edible insects in the Asia Pacific region, creating a synthesis between culture and diet practices and the concern to transition to more environmentally sustainable food production systems.

The “European Green Deal. Farm to Fork” (2020) of the European Union is an example of a strategy for food sustainability through a fair, healthy and environmentally friendly food system, generating benefits for producers, consumers, environment and climate.

The alternatives to meat and dairy products can contribute to the protection of natural resources and to economic sustainability and are adjusted to a culture of animal protection compared to the practices most used today. At the same time, some thinking has to be done about the usage of those food products and the technology involved in their production. As an example, the development of meat-like products has to be analyzed in terms of the real change in diet attitude. Will products that mimic meat be seen as transitional products to a real plant-based diet? Are they culturally similar products in that transition? Are they just pleasure products mimicking the sensory characteristics of meat?

Challenges to society and the food industry are inevitable. These include the efficiency of industrial procedures and consequent cost for consumers, the emergence of innovative new processes, the diversification of available analogues in the market, the regulatory framework for restaurants and food delivery, sustainable practices in the supply-chain, profitable return of economic investment and toxicological surveillance and safety.

The food industry may face some other challenges. The openness to meat-alternative products may be improved by tasty food products, leading to optimizing existing technologies and innovation to improve the organoleptic properties and nutritional composition of meat analogues. Another challenge is to manage the quality of information presented to the consumer. The information must be objective for a conscious and knowledgeable food choice and realistic as to the real contribution to the consumer’s health and wellbeing. It is also needed to encourage consumers to really reduce the consumption of animal products. Finally, there is a challenge to create incentives for consumers to consider integrating meat alternatives products into their diet, which might include the cost of acquisition of these food products.

## 5. Conclusions

The food industry, in response to growing consumers’ concerns about environmental sustainability, public health and ideological nature, has been making progress with the development of an increasingly diverse set of meat and dairy alternatives, such as meat analogues and plant-based drinks. To pursue this purpose, technological processes are being studied and improved. However, despite the evolution, technological approaches need to be optimized to improve cost-effectiveness, reinforce the environmental and sustainable viability of high-quality plant-based protein ingredients and increase acceptance of those food products by the consumers. The investment to be made by the industry needs to be supported and reinforced by an investment in social and cultural change towards consumer acceptance of food alternatives to meat and dairy products.

When comparing the different plant-based categories, it can be underlined that it is possible to use protein from plant sources and produce alternative protein-rich food products, guaranteeing the necessary balance between environmental sustainability, animal welfare, functional nutrition and human health and wellbeing. For a more comprehensive consumption of alternatives to meat and dairy products, continuous work will be crucial to encourage consumers to opt for these products, progressively replacing products of animal origin and their derivatives.

## Figures and Tables

**Table 1:** Main categories of alternative products to animal origin, 2014–2021.

Year	Main Food Categories	Source
2014	Textured vegetable protein	[13]
2015	Soybeans (tofu, tempeh), seitan, pulses, oilseeds, cereals and mycoprotein	[14]
2015	Soy (tofu, tempeh), seitan, rice-based products, seaweed, lupine fiber and mycoprotein	[15]
2016	Soybean (tofu, tempeh), seitan, lupine fiber, rice-based products, seaweed and Quorn	[16]
2018	Soy, gluten, pulses and oilseeds	[17]
2019	Soy, gluten, rice, oats, peas, lentils, lupine, chickpeas, mung bean and mycoprotein	[18]
2019	Soy, wheat and peas	[19]
2020	Soy, gluten, peas, mung bean protein and rice	[20]
2020	Soy, wheat, rice, peas, chickpeas, canola and rapeseed	[21]
2020	Soy (tofu, tempeh) and seitan	[22]
2020	Soy (tofu, tempeh), Quorn and artificial meat	[23]
2020	Soybean, wheat, peas, lupine, rice, potatoes and microalgae	[24]
2021	Soy, wheat, peas and mycoprotein	[25]
2021	Soy, gluten, peas, lentils, chickpeas, rice, quinoa, buckwheat, seeds and nuts	[26]
2021	Soy (tofu, yuba, tempeh, textured soy protein) and gluten	[27]

**Table 2:** Meat alternatives for human consumption (adapted from [29]).

Product	Definition and/or Sources	Benefits	Drawbacks	References
Plant-based proteins	Vegetable proteins: soy (tofu, tempeh, textured soy protein); gluten (seitan); legumes (peas, lentils, lupine, chickpeas); seeds (rapeseed, canola)	Perception of being healthier and more sustainable than meat. Greater acceptance when similar to meat. More familiar to consumers compared to mycoprotein and artificial meat. Products with the lowest environmental impact within the options presented.	Meat consumption is a common habit, and the possibility of a paradigm shift is low. The organoleptic properties motivate resistance to consumer acceptance. Distribution of products on marketplace. Possibility of banning the use of the terminology “meat” in analogue products.	[14,17,30,31,32,33,34,35,36,37,38,39,40]
Mycoprotein (fungal protein)	Product obtained through the fermentation of the fungus *Fusarium venenatum*.	Land use is lower than that used in the production of conventional animal products.	Significant impact on global warming.	[41,42]
Artificial meat	Meat produced by growing animal cell cultures.	Product that has the greatest resemblance to the original meat. Perceived as more efficient compared to conventional practices: lower resources needed per unit of meat.	Perception of being an artificial product, which raises doubts about its safety. Higher CO_2_ (carbon dioxide) emissions than meat, inefficient use of water resources and considerable expenditure on raw materials. Requires a review of food regulations.	[43,44,45,46,47,48]
Vegetable drinks	Water-soluble extracts of plant material decomposed and extracted in water for further homogenization: legumes (soybean, chickpeas); cereals (oats, rice); pseudo-cereals (quinoa, teff, amaranth); dried fruit (almond, walnut, coconut, cashew, hazelnut); seeds (sesame, sunflower).	Perception of being more sustainable. Fermentation can improve nutritional (bioavailability) and sensory properties.	Tasteless when not flavored. Concern about added sugars and sweeteners. Regulatory requirements prohibiting the use of the terminology “milk” in this type of product. Almond drink has a higher environmental impact than cow’s milk, due to the consequences of irrigation.	[49,50,51,52,53,54,55,56,57,58,59,60]
Cheese analogues	Products derived from cow’s milk that are partially or completely replaced by products of plant origin. Proteins (peanuts or soy); fats (soy, coconut, tapioca, nutritional yeast, nuts).	High-quality protein when soy is used. Possibility of altering the lipid profile, reducing the content of saturated fats. Longer validity. Lower cost when using products of lower commercial value.	Some products do not match the nutritional properties of common cheeses. The palm oil used in these products may come from unsustainable sources. Some products contain a high content of saturated fat from coconut and palm oil.	[61,62,63]
Fish analogues	Products, ingredients, or combination of ingredients used as a substitute for fish: soy, gluten, algae, mushrooms and vegetables.	Helps avoid overfishing.	Most alternatives are nutritionally deficient in proteins and essential fats (EPA and DHA).	[64,65]
Egg analogues	The ingredient responsible for the semi-solid texture of the cooked “egg” is mung bean protein. The yellowish color of these products comes from curcumin from turmeric and carotenoids from carrots.	Lower saturated fat content. Perceived as sustainable and ethical.	Highly processed. Higher content of total fat, salt and carbohydrates in comparison with eggs. More caloric. Lower quality of proteins.	[66,67]
Algae	Products rich in proteins, carbohydrates, lipids and other bioactive compounds. Some examples: *Chlorella* spp., *Arthrospira* spp., *Schizochytrium* spp.	Source of EPA (eicosapentaenoic acid) and DHA (docosahexaenoic acid). It does not need arable land. Helps to fix CO_2_ (carbon dioxide).	Regulatory problems if GMOs (genetically modified organisms) are used to improve the composition of products. Acceptance may be low due to the marine flavor.	[64,68,69]
Insects	Product rich in proteins, with essential amino acids in their composition.	Insects are one of the most abundant living species in the world. Alternative protein source to sustainable meat.	Repulsion in consumption due to the negative perception of insects.	[70,71,72,73,74]

**Table 3:** Technological processes to produce meat analogues.

Technology	Protein Sources	Synthesis	Limitations	References
Wet spinning	Soybeans, peas and fava beans	A protein solution is extruded into a coagulation bath, containing a solvent, which reduces the solubility of the protein or promotes cross-linking and fiber formation. The action of the solvent causes the protein precipitation, and together with the shear forces suffered on the nozzle, causes the proteins to align to form stretched filaments. To promote cross-linking, the solvent must contain elements such as Ca^2+^ or provide an environment that promotes the formation of intermolecular and intramolecular bonds between protein chains. The fibrous material (20 µm) formed is separated from the solvent and washed.	Due to the use of many chemical reagents, this technique generates large amounts of waste, which in turn limits its use.	[10,27,75,76]
Electrospinning	Whey, collagen, egg and soy	Technique for producing fibers with diameter in nanometer scale through high voltage. The protein solution is pushed through a nozzle and electrically accelerated by the electrical potential gradient with respect to the grounding electrode. The jet that emerges from the nozzle extends into a fine fiber (≈100 nm) while the solvent evaporates and is collected in the collector.	Requirements for the use of this technique are generally not met by plant proteins. For electrospinning to occur, proteins must be in an unfolded or intrinsically unstructured arrangement, rather than a globular structure. Plant proteins are usually globular in their native state, having to be unfolded, usually using heating before electrospinning, preventing the formation of insoluble aggregates.	[10,77,78,79,80]
Extrusion	Soy and peanuts	Most common technique for transforming proteins, particularly of plant origin. Extrusion can be classified into low moisture extrusion (<30%) that is mainly intended for the production of textured vegetable protein, while high moisture extrusion (>50%) is used to produce whole-muscle meat texture, characterized by fibrous and anisotropic structure. Other factors can influence the final product, such as: extrusion temperature, screw speed, extrusion pressure, energy input and die geometry.	Intensive energy requirements. Whether materials/ingredients can be extruded depends on the ratio of soluble and insoluble components; too many insoluble components disturb protein cross-linking and result in incoherent products.	[10,20,27,81,82,83,84,85,86]
Mixture of proteins and hydrocolloids	Soy, rice, corn and lupine	Fibrous products can be obtained by mixing proteins with hydrocolloids that precipitate with multivalent cations. After mixing, the fibrous products are washed and the excess water is removed by pressing, resulting in dry matter contents between 40% and 60%. In this process, various combinations of hydrocolloid proteins and multivalent cations can be used, such as casein and alginate.	Intensive use of resources. Despite the initial ordering in the shear direction, the subsequent steps destroy this large range ordering, limiting the use of minced meat products such as burgers and schnitzels.	[10,87]
Freeze structuring	Plant proteins	In freeze structuring or freeze alignment, the aqueous solution (protein paste) is frozen to be structured. Removal of heat from a well-mixed slurry gives an isotropic structure, but when heat is removed unidirectionally without mixing, the alignment of the ice crystal needles produces anisotropic structures. Needle size must be adapted to temperature and freezing rate. Subsequently, the frozen product is dried without melting the ice crystals, for example, by lyophilization, to obtain a porous microstructure with an orientation parallel to the proteins.	To obtain distinct fibrous products, the proteins should have relatively good solubility prior to freezing, and during the freezing process, these proteins become insoluble.	[10,88,89,90]
Shear cell technology	Soy and gluten	Based on the recognition that extrusion is an effective process, but not properly defined, a technology based on shear flow deformation was created. The final structure obtained with this technique depends on the ingredients and processing conditions. Fibrous products are obtained with calcium caseinate and various vegetable protein blends such as soy protein concentrate, soy protein isolate, wheat gluten and pectin.	The mechanisms underlying this process are not well understood.	[91,92,93,94,95,96]
3D printing	Algae	Three-dimensional food printing is rapidly developing with various 3D printing techniques available. The most common is based on syringe injection. In this process, a protein solution with a high viscosity is extruded through a thin syringe nozzle and moved layer by layer to form a 3D product (for example, a muscle-shaped structure). Printing is based on a pre-engineered digital template and 3D printing models must withstand cooking processes. Printability refers to physical and chemical properties, ensuring its fluidity out of the nozzle and the ability to maintain and quickly harden the post-layout 3D structure.	Restriction of food materials that can be printed directly. The printed protein solution must be homogeneous and have adequate printability. When ordinary foods are changed by 3D printing, food loses some nutritional value and sensory qualities. Lack of research in 3D printing of functional foods.	[20,97,98,99,100,101,102]

**Table 4:** Main sources of plant-based proteins.

Food Product	Characterization and Production
Soy—Conventional products	
Tofu	Tofu is produced from “milk” from soybeans ground in hot water, after being properly peeled. After heating, the hard parts (*Okara*) are separated from the “milk”, and the protein is coagulated through the addition of a coagulant (*nigari*, magnesium sulfate or calcium chloride) [104].
Tempeh	In the production of tempeh (paste from fermented soybeans), soybeans are peeled, soaked and cooked. Subsequently, they are cooled and inoculated with a mold (*rhizopus*), which makes the preparation ferment [104].
Miso	Miso is a fermented soybean paste, produced from cooked soybeans and mixed with other cereals, which gives the miso paste variability *(miso hatcho, miso mugi, miso genmai*). After the fermentation of the grains, the mixture is salted, obtaining a thick and nutritious paste that contains live bacteria and ferments [104].
Soybean—Utility in the production of meat analogues	
Soy flour, soy protein concentrate and soy protein isolate	Soy ingredients are the most commonly used in meat analogues due to their functional properties such as water holding ability, gelling, fat absorption and emulsifying ability [17]. Soy protein isolate stands out for its high protein purity, light color and mild flavor compared to other soy ingredients [17].
Other Legumes	
Lentils, peas and chickpeas	Protein source (15% to 40%), essentially lysine. Air-classification is an extraction process that adapts to the characteristics of peas and lentils (wide diameter and uniform distribution of starch) [105]. Alkaline extraction followed by isoelectric precipitation is considered the most common method in the extraction of vegetable proteins, due to its simplicity and production of concentrates with high protein purity. Other methodologies used are alkaline extraction followed by ultrafiltration, aqueous extraction and saline extraction [106].
Lupine	The technological challenges to optimize the production and processing of lupine protein are related to the maintenance of lupine oil and fiber, due to the potential that fiber demonstrates in functional foods, with oil being an attractive product due to its balanced composition of fatty acids and their bioactive lipid content [107,108] Given the high protein content, lupine is considered a great raw material and can be used as an egg substitute in the production of cakes and bread [109].
Other Legumes—Utility in the production of meat analogues	
Peas, lentils, lupines and chickpeas	The functional properties (emulsification, stabilization and gel formation) of these legumes were studied, and it was concluded that [110,111,112,113,114,115,116,117,118,119]: Among them, the most promising for the production of meat analogues was pea protein, which, in the study, was structured by high moisture extrusion; Chickpeas, lentils and lupines showed good emulsifying, foaming and stabilizing capacity; Apart from chickpeas, these proteins have weaker gelling abilities than soybeans.
Gluten	
Seitan	It is produced by preparing wheat flour, as in the production of bread dough. This mass is washed in a colander with running water. In this process, fats and carbohydrates are removed. The washed pasta is cooked with soy sauce (*shoyu* or *tamari*) and thus gains a hard consistency [104].
Gluten—Utility in the production of meat analogues	
Gluten	Gluten is one of the main ingredients for the formation of fibrous structures, so it is common to be present in the composition of meat analogues [120].

**Table 5:** Production stages of plant-based drinks. Adapted from [123].

Process	Consideration	Limitations	Reference
Roasting	Used in peanut, sesame and hazelnut drinks; Roasting increases emulsion stability and protein solubility; It can reduce acidity, total solids, protein and fat, and avoid bitterness.	Roasting reduces acidity, total solids, protein and fat.	[124,125,126,127]
Dry grinding	It is not the most recommended process; Wet grinding is an alternative to dry grinding.	High energy consumption. Higher requirements of control compared to wet grinding.	[128]
Peeling	Use of acids or bases. Using citric acid (2% concentration at 90 °C in 2 min), the nut is peeled; The base commonly used is sodium hydroxide (NaOH); Use of water is feasible, and the process takes longer (18 to 20 h). The time depends on the raw material used. A subsequent wash should be carried out to remove traces of the used acid or base. The peeling allows to remove the toxic components present in the skin, removing the bitter taste.	Inorganic chemical compounds must be used (e.g., sodium hydroxide), increasing the water consumption and the amount of wastewater to be treated.	[129,130,131,132,133,134]
Soaking in water	Used for soybeans, hazelnuts, rice, sesame, peanuts and almonds; Hydration (soaking) and softening of raw materials take place. Toxins and nutrients are released into the water.	Time-consuming operation (up to 24 h). Water consumption.	[50,122,125,132,135,136,137,138,139]
Blanching	Used for soybeans, almonds, coconut, sesame, peanuts, rice and quinoa; Decreases microbial load; Inactivates enzymes; Steam blanching can be used (increases total solids and protein yield).	Amount of wastewater to be treated.	[122,134,139,140,141,142,143,144,145,146,147]
Wet milling	Applied to soybeans, coconut, cashew nuts, hazelnuts, hemp seeds, almonds, walnuts and peanuts; The amount of water added, the grinding temperature, the pH and the type of grinding are some of the factors that affect the final product.	Water consumption.	[121,127,129,132,140,141,148,149,150,151]
Filtration	It is applied to separate the liquid from the solid phase (cake) of the ground raw material; Filtration with double layer gauze, muslin cloth (25 μm) or filter paper of different sizes can be used; Ultrafiltration is also used (hazelnut, sesame and corn).	Ultrafiltration can be an expensive operation.	[124,129,131,132,134,139,141,142,150,152,153,154,155,156]
Addition of ingredients	In industry, sunflower lecithin and locust bean gums and gellan are used to increase the stability of solutions; Xanthan gum is commonly used as a thickener and stabilizer; Ascorbic acid is added to prevent oxidation; Sweeteners (sugarcane, sugar syrup, sucrose) and sea salt are incorporated to improve the flavor of the preparation (some varieties may contain vanilla or cocoa); To improve the silky appearance, sunflower oil and olive oil are used.	Some components can cause allergic reactions. Increase the cost of the final product.	[121,122,130,137,139,146,147,157,158]
Fortification and enrichment	During production, different compounds are incorporated to increase the nutritional and organoleptic properties of the final product; To increase the protein content, lentils can be used; Calcium and vitamins (A, B2, B1, B12, D2 and E) are also added to increase vitamin and mineral content; Calcium citrate is used to increase the amount of calcium in the final product.	Some components can cause allergic reactions. These components are not naturally present.	[50,159]
Homogenization	It aims to improve the stability of the product; At this stage, the temperature of the product can increase between 5 °C and 10 °C.	Raising product temperature.	[127,130,138,160,161]
Sterilization	Objective of increasing the shelf life of the product; Pasteurization and sterilization can be applied.	Negative effects of temperature on nutritional and sensorial quality of products.	[121,138,162,163,164]
Aseptic packaging and cold storage	Keep the lifetime of the product; The storage temperature must be +4 °C.	Increase the chance of physical damage in the final product.	[123]

**Table 6:** Ingredients used in cheese analogue production (adapted from [182]).

Ingredient	Function	Example
Fat	Desired composition and texture	Butter, soy, corn
Milk proteins	Desired composition and texture	Casein, whey, caseinates
Vegetable proteins	Desired composition, lower price relative to casein	Peanuts and gluten
Starch	Casein substitute (lower price)	Rice, potato, natural and modified corn
Hydrocolloid stabilizers	Desired texture and stability	Sodium phosphate, sodium citrate, guar gum, xanthan gum
Acidifying agents	pH control	Organic acids, lactic, citric and phosphoric acid
Flavorings	Desired flavor	Smoked extract, spices, cheese-modifying enzyme
Flavor enhancers	Desired flavor	Salt and yeast extract
Dyes	Desired color	Paprika, annatto and artificial dyes
Preservatives	Shelf-life extension	Nisin, potassium sorbate, calcium sodium propanoate

**Table 7:** Edible insects for human food intake (adapted from [73]).

Order	Common Name
Coleoptera	Beetles
Lepidoptera	Butterflies
Hymenoptera	Ants, wasps and bees
Hemiptera	Cicadas
Diptera	Flies
Odonata	Dragonflies
Isopters	Termites
Orthopterans	Locusts and crickets

## Data Availability

Not applicable.
